# Diagnosis of fracture-related infection in patients without clinical confirmatory criteria: an international retrospective cohort study

**DOI:** 10.5194/jbji-8-133-2023

**Published:** 2023-04-21

**Authors:** Niels Vanvelk, Esther M. M. Van Lieshout, Jolien Onsea, Jonathan Sliepen, Geertje Govaert, Frank F. A. IJpma, Melissa Depypere, Jamie Ferguson, Martin McNally, William T. Obremskey, Charalampos Zalavras, Michael H. J. Verhofstad, Willem-Jan Metsemakers

**Affiliations:** 1 Trauma Research Unit, Department of Surgery, Erasmus MC, University Medical Centre Rotterdam, Rotterdam, the Netherlands; 2 Department of Trauma Surgery, University Hospitals Leuven, Leuven, Belgium; 3 Department of Development and Regeneration, KU Leuven, Leuven, Belgium; 4 Department of Trauma Surgery, University Medical Centre Groningen, Groningen, the Netherlands; 5 Department of Trauma Surgery, University Medical Centre Utrecht, Utrecht, the Netherlands; 6 Department of laboratory medicine, University Hospitals Leuven, Leuven, Belgium; 7 Department of Microbiology, Immunology and Transplantation, Laboratory of Clinical Bacteriology and Mycology, KU Leuven, Leuven, Belgium; 8 The Bone Infection Unit, Nuffield Orthopaedic Centre, Oxford University Hospitals, Oxford, UK; 9 Department of Orthopaedic Surgery and Rehabilitation, Vanderbilt University Medical Center, Nashville, Tennessee, USA; 10 Department of Orthopaedic Surgery, Keck School of Medicine, University of Southern California, Los Angeles, USA

## Abstract

**Background**: fracture-related infection (FRI) remains a serious complication in orthopedic trauma. To standardize daily clinical practice, a consensus definition was established, based on confirmatory and suggestive criteria. In the presence of clinical confirmatory criteria, the diagnosis of an FRI is evident, and treatment can be started. However, if these criteria are absent, the decision to surgically collect deep tissue cultures can only be based on suggestive criteria. The primary study aim was to characterize the subpopulation of FRI patients presenting without clinical confirmatory criteria (fistula, sinus, wound breakdown, purulent wound drainage or presence of pus during surgery). The secondary aims were to describe the prevalence of the diagnostic criteria for FRI and present the microbiological characteristics, both for the entire FRI population. **Methods**: a multicenter, retrospective cohort study was performed, reporting the demographic, clinical and microbiological characteristics of 609 patients (with 613 fractures) who were treated for FRI based on the recommendations of a multidisciplinary team. Patients were divided in three groups, including the total population and two subgroups of patients presenting with or without clinical confirmatory criteria. **Results**: clinical and microbiological confirmatory criteria were present in 77 % and 87 % of the included fractures, respectively. Of patients, 23 % presented without clinical confirmatory criteria, and they mostly displayed one (31 %) or two (23 %) suggestive clinical criteria (redness, swelling, warmth, pain, fever, new-onset joint effusion, persisting/increasing/new-onset wound drainage). The prevalence of any suggestive clinical, radiological or laboratory criteria in this subgroup was 85 %, 55 % and 97 %, respectively. Most infections were monomicrobial (64 %) and caused by *Staphylococcus aureus*. **Conclusion**: clinical confirmatory criteria were absent in 23 % of the FRIs. In these cases, the decision to operatively collect deep tissue cultures was based on clinical, radiological and laboratory suggestive criteria. The combined use of these criteria should guide physicians in the management pathway of FRI. Further research is needed to provide guidelines on the decision to proceed with surgery when only these suggestive criteria are present.

## 1 Introduction

Fracture-related infection (FRI) remains a serious complication related to orthopedic trauma. It is associated with an increased morbidity, potentially leading to loss of function or even amputation of the affected limb (Metsemakers et al., 2017b). The treatment often consists of multiple surgeries and hospital admissions, which significantly increase healthcare costs (Metsemakers et al., 2017a; Iliaens et al., 2021).

Until recently, both clinical practice and research were hampered by the lack of a universally accepted definition of FRI. A systematic review investigating the definitions used in clinical trials to describe infectious complications after fracture fixation found that 70 % of the included studies did not mention a definition for FRI. Only 2 % of the articles referred to the Centers for Disease Control and Prevention criteria for surgical site infection (Metsemakers et al., 2018a). However, although these criteria have been validated for the surveillance of surgical site infections, their diagnostic value for FRI seems to be limited (Sliepen et al., 2021).

With the intention of improving the quality of published research and standardizing clinical practice, a consensus definition was established. The diagnostic criteria were first published in 2018 (Metsemakers et al., 2018b) and updated by adding histopathology and nuclear imaging in 2020 (Govaert et al., 2020). The foundation of the consensus definition is that some criteria are pathognomonic for infection (confirmatory criteria), while others are less specific and might also be present in the absence of infection (suggestive criteria) (Table 1) (Metsemakers et al., 2018b; Govaert et al., 2020).

The confirmatory criteria were recently validated in a large retrospective cohort study (Onsea et al., 2022). The presence of at least one confirmatory criterion was associated with a sensitivity of 97.5 % and a specificity of 100 % (Onsea et al., 2022). While the diagnosis of infection is thus clear in the presence of clinical confirmatory criteria and treatment can immediately be started, this is less evident in the absence of these criteria. In these patients, the decision to operate and collect deep tissue samples can only be based on a combination of clinical, radiological and laboratory suggestive criteria (Metsemakers et al., 2018b; Govaert et al., 2020).

The primary aim of this study was to characterize the subpopulation of FRI patients presenting without clinical confirmatory criteria. Secondary aims were to describe the prevalence of the diagnostic criteria for FRI and present the microbiological characteristics, both for the entire FRI population. The study presented is a secondary analysis combining previously published data (Sliepen et al., 2021; Onsea et al., 2022) with new data from two additional hospitals.

**Table 1:** Diagnostic criteria for fracture-related infections (Metsemakers et al., 2018b; Govaert et al., 2020).

Confirmatory criteria
Clinical criteria
– Fistula, sinus or wound breakdown (with communication to the bone or implant)
– Purulent drainage from the wound or presence of pus during surgery
Microbiological criteria
– Phenotypically indistinguishable microorganisms isolated from at least two separate deep tissue or implant specimens
Histopathology criteria
– Presence of microorganisms in deep tissue specimens
– Presence of at least five polymorphonuclear neutrophils per high-power field
Suggestive criteria
Local clinical criteria
– Redness
– Swelling
– Warmth
– Pain (without weight bearing, increasing over time, new onset)
Systemic clinical criteria
– Fever (oral temperature measurement of ≥38.3 ${}^{\circ }$ C (101 ${}^{\circ }$ F))
Other clinical criteria
– New-onset joint effusion
– Persistent, increasing or new-onset wound drainage, beyond the first few days postoperatively,
without solid alternative explanation
Radiological criteria
– Conventional radiology, CT, MRI
Nuclear imaging criteria
– WBC scan, ${}^{18}$ F-FDG-PET, bone scintigraphy
Laboratory criteria
– Elevated serum inflammatory markers (WBC count, CRP and/or ESR)
Microbiological criteria
– Pathogenic organism identified by culture from a single deep tissue or implant specimen

CRP – C-reactive protein, CT – computed tomography, ESR – erythrocyte sedimentation rate, ${}^{18}$ F-FDG-PET – fluorodeoxyglucose positron emission tomography, MRI – magnetic resonance imaging, WBC – white blood cell.

## 2 Materials and methods

### 2.1 Ethics approval

The study protocol was approved by the Ethics Committee of the University Hospitals Leuven, Belgium (Ethics Committee Research UZ/KU Leuven; S62394) and conducted following good clinical practice guidelines. A data-sharing agreement was signed between participating centers.

### 2.2 Study design

This is a secondary analysis of data including patients of a previous retrospective study that was aimed at validating the diagnostic criteria for FRI (Onsea et al., 2022). For this study, the dataset was expanded with patients treated in two additional hospitals. The hospitals were based in Belgium (University Hospitals Leuven), the Netherlands (University Medical Centre Rotterdam, University Medical Centre Groningen, University Medical Centre Utrecht), the United Kingdom (Oxford University Hospitals) and the United States (Vanderbilt University Medical Center), and they all serve as a tertiary referral center for FRI. Patients between January 2015 and November 2019 were included based on an “intention to treat” as recommended by a multidisciplinary team, consisting of surgeons, infectious disease specialists, microbiologists, radiologists and clinical pharmacists. Patients with fractures of the hand, skull or spine and patients with pathological fractures were excluded.

### 2.3 Data collection

Medical records were reviewed, and patient demographics including sex, age, body mass index (BMI) and American Society of Anesthesiologists (ASA) score were documented. The collected data related to the fracture included localization, Gustilo–Anderson (GA) type and time from primary fracture management to the onset of symptoms. All confirmatory and suggestive criteria of the FRI consensus definition were recorded. Lab values were considered elevated when there was >5 mg L ${}^{-1}$ for C-reactive protein (CRP), $>10\times 10^{9}$ L ${}^{-1}$ for white blood cell (WBC) count and >20 mm h ${}^{-1}$ for erythrocyte sedimentation rate (ESR). Quantitative histopathology was excluded when performed within 2 months from the primary fracture treatment. All pathogens were recorded when present in two separate deep tissue cultures. The presence of a pathogen in a single deep tissue culture was only recorded when a virulent pathogen was isolated. Virulent pathogens were defined a priori based on a high likelihood of causing disease, the low probability of these being present as contaminants and the clinical experience of infectious disease physicians. This group included Gram-negative Bacilli, *Staphylococcus aureus*, *Staphylococcus lugdunensis*, Enterococci, beta-hemolytic Streptococci, milleri group Streptococci, *Streptococcus pneumoniae* and *Candida* species (Dudareva et al., 2018). Single positive cultures with non-virulent pathogens were considered contaminants and not further evaluated.

### 2.4 Statistical analysis

Statistical analysis was performed using SPSS for Windows (SPSS, Chicago, Illinois, USA). Normality of continuous data was tested using the Shapiro–Wilk test. The prevalence of diagnostic criteria was presented using frequencies and percentages. Continuous data, which were all non-parametric, are presented as median ( $P_{25}$ – $P_{75}$ ). Data are reported for the entire study population and the subgroups of patients presenting with or without clinical confirmatory criteria. Categorical variables were compared using the chi-square test or Fisher's exact test, as appropriate.

## 3 Results

### 3.1 Patient demographics and fracture characteristics

During the study period, 609 patients were included. Four of these sustained FRIs at different anatomical locations at different time points. The total number of included infections was therefore 613. An overview of patient characteristics is provided in Table 2. The majority of patients were male ( n=427 ; 70 %), and 119 (19 %) patients were polytrauma cases. Half of the study population ( n=304 ; 50 %) presented with mild systemic disease (ASA 2). Severe systemic disease (ASA 4) was only present in 11 (2 %) patients. Fractures of the tibia/fibula were most prevalent ( n=33 8; 55 %), followed by fractures of the femur ( n=98 ; 16 %). One-third ( n=203 ; 33 %) of the fractures were open, of which the majority ( n=103 ; 51 %) were GA type III. In most cases ( n=407 ; 66 %), a plate and screw osteosynthesis was performed to treat the initial fracture, followed by intramedullary nailing ( n=145 ; 24 %).

**Table 2:** Patient characteristics. Statistically significant p values are displayed in bold.

	All		Without clinical		With clinical		p value
			confirmatory criteria		confirmatory criteria		
	N ${}^{*}$	N (%)	N	N (%)	N	N (%)	
Age (years)	609	50 (38–63)	143	52 (40–60)	466	50 (37–63)	0.774
Sex (male)	609	427 (70 %)	143	96 (67 %)	466	331 (71 %)	0.404
BMI (kg m ${}^{-2}$ )	604	26.3 (23.2–30)	142	26.4 (23.9–30)	462	26.3 (23–30)	0.356
ASA	609		143		466		**0.012**
1		147 (24 %)		37 (26 %)		110 (24 %)	
2		304 (50 %)		69 (48 %)		235 (50 %)	
3		147 (24 %)		30 (21 %)		117 (25 %)	
4		11 (2 %)		7 (5 %)		4 (1 %)	
Smoking status	613		144		469		0.353
Active		184 (30 %)		37 (26 %)		147 (31 %)	
Past		91 (15 %)		21 (15 %)		70 (15 %)	
Never		328 (54 %)		85 (59 %)		243 (52 %)	
Not specified		10 (2 %)		1 (1 %)		9 (2 %)	
Fracture localization	613		144		469		< **0.001**
Humerus		49 (8 %)		12 (8 %)		37 (8 %)	
Clavicle		19 (3 %)		5 (4 %)		14 (3 %)	
Radius or ulna		43 (7 %)		5 (4 %)		38 (8 %)	
Femur		98 (16 %)		42 (29 %)		56 (12 %)	
Tibia and/or fibula		338 (55 %)		69 (48 %)		269 (57 %)	
Foot		25 (4 %)		4 (3 %)		21 (5 %)	
Pelvic ring or acetabulum		30 (5 %)		4 (3 %)		26 (6 %)	
Patella		6 (1 %)		2 (2 %)		4 (1 %)	
Rib or sternum		4 (1 %)		1 (1 %)		3 (1 %)	
Scapula		1 (0 %)		0 (0 %)		1 (0 %)	
Type of fixation	613		144		469		0.125
Plate and screw osteosynthesis		407 (66 %)		85 (59 %)		322 (69 %)	
Intramedullary nail		146 (24 %)		47 (33 %)		99 (21 %)	
Screw osteosynthesis		10 (2 %)		1 (1 %)		9 (2 %)	
Pinning/cerclage		21 (3 %)		5 (3 %)		16 (3 %)	
Definite external fixator		20 (3 %)		5 (3 %)		15 (3 %)	
Plate and screw osteosynthesis and nail		4 (1 %)		0 (0 %)		4 (1 %)	
None		5 (1 %)		1 (1 %)		4 (1 %)	
Primary external fixation	613	177 (29 %)	144	43 (30 %)	469	134 (29 %)	0.754
Open fracture	613	203 (33 %)	144	43 (29.9 %)	469	160 (34 %)	0.364
GA type I	203	37 (18 %)	43	6 (14.0 %)	160	31 (19 %)	0.534
GA type II	203	63 (31 %)	43	16 (37.2 %)	160	47 (29 %)	
GA type III	203	103 (51 %)	43	21 (48.8 %)	160	82 (51 %)	
Polytrauma (ISS >15 )	613	119 (19 %)	144	32 (22 %)	469	87 (19 %)	0.330
Unknown		33 (5 %)		9 (6 %)		24 (5 %)	
Time from primary fixation until onset	613	43 (16–178)	144	81 (15–293.8)	469	41 (16–124.5)	0.081
of symptoms (d)							
Antibiotic therapy less than 2 weeks	613	107 (17 %)	144	17 (11.8 %)	469	90 (19 %)	**0.045**
prior to tissue sampling							

Data are shown as median ( $P_{25}$ – $P_{75}$ ) or as N (%). ${}^{*}$ The number of patients is 609. The corresponding number of fractures is 613, because four patients sustained two FRIs at different anatomical locations. BMI – body mass index, ASA – American Society of Anesthesiologists, GA – Gustilo–Anderson.

### 3.2 The prevalence of diagnostic criteria for FRI

The prevalence of confirmatory and suggestive criteria for the total population and the subgroups presenting with or without clinical confirmatory criteria is displayed in Table 3. Clinical confirmatory criteria were present in 469 (77 %) FRIs. Of these, 139 (30 %) patients presented with a fistula, sinus or wound breakdown. Purulent drainage or pus was present in 168 (36 %) patients. The combination of both clinical confirmatory criteria was present in 162 (35 %) patients. In 536 (87 %) FRIs, the infection was confirmed based on the culture of phenotypically indistinguishable microorganisms isolated from at least two separate deep tissue specimens. Histopathological confirmation of the presence of microorganism by specific staining techniques was possible in 69 (46 %) of the 151 cases it was used in. Quantitative histopathology was performed in 79 patients. A minimum of five polymorphonuclear neutrophils (PMN) per high-power field (HPF) was found in 23 (29 %) of these. In total, at least one confirmatory criterion was present in 602 (98 %) of the infections.

**Figure 1:**
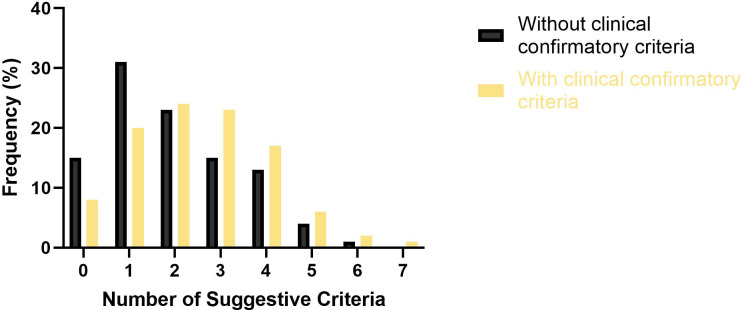
Number of clinical suggestive criteria per patient.

Of the 144 patients (23 %) without clinical confirmatory criteria, most presented with either one ( n=44 ; 31 %) or two ( n=33 ; 23 %) clinical suggestive criteria (Fig. 1). Any clinical suggestive criterion was present in 123 (85 %) patients. Pain ( n=80 ; 56 %) was the only clinical suggestive criterion that was more prevalent in the group without clinical confirmatory criteria. When excluding pain, at least one clinical suggestive criterion was present in 97 (67 %) of the patients without clinical confirmatory criteria and in 420 (90 %) of patients with clinical confirmatory criteria ( p<0.001 ). This difference was mainly expressed in redness (40 % vs. 59 %; p<0.001 ) and wound drainage (25 % vs. 50 %; p<0.001 ).

Radiological criteria on conventional radiography were more prevalent in patients without clinical confirmatory criteria (53 % vs. 37 %; p=0.003 ). Failure of progression of bone healing (34 % vs. 19 %; p=0.002 ) and implant loosening (18 % vs. 11 %; p=0.043 ) were mainly present in this subgroup. Radiological criteria on CT were present in 24 (53 %) patients without clinical confirmatory criteria. Magnetic resonance imaging (MRI) was only performed in three patients without clinical confirmatory criteria. Two of these displayed suggestive criteria for infection. Nuclear imaging was performed in 24 patients without clinical confirmatory criteria and evaluated as being positive for infection in 17 (71 %) of these. Fluorodeoxyglucose positron emission tomography ( ${}^{18}$ F-FDG-PET) most often displayed criteria of infection (71 %), followed by bone scintigraphy (69 %) and WBC scan (50 %).

The frequency of serum inflammatory marker elevation was comparable between patients presenting without or with clinical confirmatory criteria. In both groups, the presence of any laboratory criterion was 97 %. In patients without clinical confirmatory criteria, an elevated CRP was most prevalent ( n=101 , 83 %).

**Table 3:** Prevalence of FRI criteria for the total population and the subpopulation without clinical confirmatory criteria. Statistically significant p values are displayed in bold.

Diagnostic criteria	All		Without clinical confirmatory criteria		With clinical confirmatory criteria		p value
	( n=613 )		confirmatory criteria		confirmatory criteria		
			( n=144 )		( n=469 )		
	N	N (%)	N	N (%)	N	N (%)	
Confirmatory criteria							
Any confirmatory criterion ${}^{*}$	613	602 (98 %)	144	133 (92 %)	469	469 (100 %)	< **0.001**
Clinical criteria							
Fistula, sinus or wound breakdown	613	301 (49 %)	144	0 (0 %)	469	301 (64 %)	< **0.001**
Purulent drainage or pus	613	330 (54 %)	144	0 (0 %)	469	330 (70 %)	< **0.001**
Microbiological criteria							
Phenotypically indistinguishable	613	536 (87 %)	144	131 (91 %)	469	405 (86 %)	0.153
microorganisms isolated from at least two							
separate deep tissue cultures							
Histopathological criteria							
Histological presence of microorganisms	151	69 (46 %)	26	13 (50 %)	125	56 (45 %)	0.669
Histological presence of ≥5 PMNs/HPF	79	23 (29 %)	19	7 (37 %)	60	16 (27 %)	0.400
Suggestive criteria							
Clinical criteria of inflammation							
Any clinical criterion ${}^{*}$	613	553 (90 %)	144	123 (85 %)	469	430 (92 %)	**0.036**
Any clinical criterion excl. pain ${}^{*}$	613	517 (84 %)	144	97 (67 %)	469	420 (90 %)	< **0.001**
Redness rubor	613	333 (54 %)	144	57 (40 %)	469	276 (59 %)	< **0.001**
Local warmth/calor	613	128 (21 %)	144	25 (17 %)	469	103 (22 %)	0.291
Swelling/tumor	613	283 (46 %)	144	59 (41 %)	469	224 (48 %)	0.181
Pain/dolor	613	299 (49 %)	144	80 (56 %)	469	219 (47 %)	0.070
New-onset joint effusion	613	51 (8 %)	144	10 (7 %)	469	41 (9 %)	0.606
Wound drainage	613	270 (44 %)	144	36 (25 %)	469	234 (50 %)	< **0.001**
Fever ≥38.3 ${}^{\circ }$ C	613	73 (12 %)	144	14 (10 %)	469	59 (13 %)	0.462
Radiological criteria							
Any radiological criterion ${}^{*}$	538	251 (47 %)	121	67 (55 %)	417	184 (44 %)	**0.030**
Conventional radiography	494	203 (41 %)	119	63 (53 %)	375	140 (37 %)	**0.003**
Implant loosening	494	64 (13 %)	119	22 (18 %)	375	42 (11 %)	**0.043**
Bone lysis	494	83 (17 %)	119	17 (14 %)	375	66 (18 %)	0.482
Failure of progression	494	112 (23 %)	119	40 (34 %)	375	72 (19 %)	**0.002**
Sequestration	494	16 (3 %)	119	2 (2 %)	375	14 (4 %)	0.379
Periosteal bone formation	494	19 (4 %)	119	6 (5 %)	375	13 (3 %)	0.420
Implant failure	494	15 (3 %)	119	5 (4 %)	375	10 (3 %)	0.371
Abscess	494	1 (0 %)	119	0 (0 %)	375	1 (0 %)	1.000
CT scan	221	114 (52 %)	45	24 (53 %)	176	90 (51 %)	0.868
Implant loosening	221	15 (7 %)	45	3 (7 %)	176	12 (7 %)	1.000
Bone lysis	221	36 (16 %)	45	7 (16 %)	176	29 (16 %)	1.000
Failure of progression	221	65 (29 %)	45	16 (36 %)	176	49 (28 %)	0.360
Sequestration	221	17 (8 %)	45	1 (2 %)	176	16 (9 %)	0.206
Periosteal bone formation	221	7 (3 %)	45	2 (4 %)	176	5 (3 %)	0.633
Implant failure	221	3 (1 %)	45	2 (4 %)	176	1 (1 %)	0.106
Abscess	221	22 (10 %)	45	1 (2 %)	176	21 (12 %)	0.054
MRI	8	5 (63 %)	3	2 (67 %)	5	3 (60 %)	1.000
Implant loosening	8	3 (38 %)	3	1 (33 %)	5	2 (40 %)	1.000
Bone lysis	8	0 (0 %)	3	0 (0 %)	5	0 (0 %)	n/a
Failure of progression	8	1 (13 %)	3	1 (33 %)	5	0 (0 %)	0.375
Sequestration	8	0 (0 %)	3	0 (0 %)	5	0 (0 %)	n/a
Periosteal bone formation	8	0 (0 %)	3	0 (0 %)	5	0 (0 %)	n/a
Implant failure	8	1 (13 %)	3	1 (33 %)	5	0 (0 %)	0.375
Abscess	8	2 (25 %)	3	1 (33 %)	5	1 (20 %)	1.000

**Table 3:** Continued.

Diagnostic criteria	All		Without clinical confirmatory criteria		With clinical confirmatory criteria		p value
	( n=613 )		confirmatory criteria		confirmatory criteria		
			( n=144 )		( n=469 )		
	N	N (%)	N	N (%)	N	N (%)	
Nuclear imaging criteria							
Any nuclear imaging criterion ${}^{*}$	57	38 (67 %)	24	17 (71 %)	33	21 (64 %)	0.777
Bone scintigraphy	26	19 (73 %)	16	11 (69 %)	10	8 (80 %)	0.668
WBC scan	24	12 (50 %)	14	7 (50 %)	10	5 (50 %)	1.000
${}^{18}$ F-FDG-PET scan	26	17 (65 %)	7	5 (71 %)	19	12 (63 %)	1.000
Laboratory criteria							
Any laboratory criterion ${}^{*}$	453	441 (97 %)	107	104 (97 %)	346	337 (97 %)	1.000
ESR >20 mm h ${}^{-1}$	100	74 (74 %)	21	15 (71 %)	79	59 (75 %)	0.783
WBC $>10\times 10^{9}$ L ${}^{-1}$	531	219 (41 %)	121	48 (40 %)	410	171 (42 %)	0.753
CRP >5 mg L ${}^{-1}$	525	423 (81 %)	121	101 (83 %)	404	322 (80 %)	0.432
Microbiological criteria							
Single positive culture	77	30 (39 %)	13	6 (46 %)	64	24 (38 %)	0.756

${}^{*}$ The criterion was scored as “present” if any one of the mentioned criteria was present, while it was scored as “absent” if none of the mentioned criteria were present. CRP – C-reactive protein, CT – computed tomography, ESR – erythrocyte sedimentation rate, ${}^{18}$ F-FDG-PET – fluorodeoxyglucose positron emission tomography, HPF – high-power field, MRI – magnetic resonance imaging, n/a – not applicable, PMN – polymorphonuclear neutrophil, WBC – white blood cell.

### 3.3 Microbiological criteria and epidemiology

In 536 (87 %) of the FRIs, phenotypically indistinguishable microorganisms were isolated from at least two separate deep tissue specimens. A single positive culture with a virulent microorganism was found in 30 (5 %) of the infections. Of the remaining 47 FRIs (8 %) presenting without positive cultures, 19 (40 %) were treated with antibiotics in the 2 weeks prior to tissue sampling.

In the subgroup of patients without clinical confirmatory criteria, infection could be confirmed in 131 (91 %) FRIs through microbiological sampling. Single positive cultures with a virulent microorganism and negative cultures were found in, respectively, six (4 %) and seven (5 %) patients of this subgroup.

**Figure 2:**
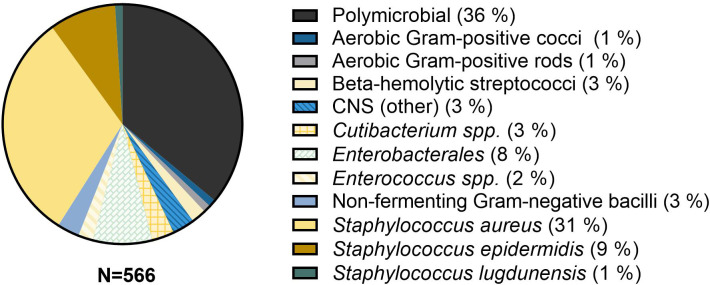
Microbiological epidemiology. CNS – coagulase-negative Staphylococci, spp. – species (not further specified).

Of the 566 culture positive FRIs, 361 (64 %) were monomicrobial. In these monomicrobial infections, *Staphylococcus* *aureus* (31 %) was the most frequently cultured pathogen, followed by *S. epidermidis* (9 %) and Enterobacterales (8 %) (Fig. 2).

## 4 Discussion

While FRI remains a challenging complication in orthopaedic trauma care, the development of an international consensus definition has expedited advances in both clinical practice and research. The definition was composed to include both confirmatory and suggestive criteria of infection (Metsemakers et al., 2018b; Govaert et al., 2020). This secondary analysis on data from a multicenter, multinational, retrospective cohort study assesses the prevalence of the FRI consensus criteria in 609 patients and performs an in-depth analysis of the subpopulation of FRI patients presenting without clinical confirmatory criteria.

### 4.1 Diagnostic criteria for FRI

The diagnostic approach to FRI depends on the presence or absence of confirmatory criteria. These criteria were validated in a recent paper by Onsea et al. (2022). In the presence of clinical confirmatory criteria, such as a fistula/sinus/wound breakdown or purulent drainage from the wound, the diagnosis is immediately confirmed, and treatment should be started (Govaert et al., 2020). In our cohort, any of these clinical confirmatory criteria was present in 469 (77 %) of the infected fractures.

When the clinical confirmatory criteria are not present, FRI can still be confirmed through microbiological and histopathological criteria. In a study by Dudareva et al. (2021), the presence of the same pathogen in at least two out of five deep cultures was associated with a high diagnostic accuracy. In the subgroup of patients without clinical confirmatory criteria in our cohort, this microbiological confirmatory criterion was present in 131 of 144 (91 %) FRIs. A single positive culture with a virulent microorganism was found in another six (4 %) fractures. In the recent validation study, this criterion was associated with a specificity of 100 % (Onsea et al., 2022). Negative cultures were found in seven (5 %) patients of this subgroup. This number is in line with previous research, showing a negative culture rate of 6 % in implant-associated infections (Stephan et al., 2021).

Histopathological confirmation by the presence of visible microorganisms in deep tissue was included in the original FRI definition (Metsemakers et al., 2018b). In the revised edition, the presence of more than five PMNs/HPF was included as a confirmatory criterion in chronic/late-onset infections (Govaert et al., 2020). The role of this criterion was investigated in previous research, demonstrating a sensitivity and specificity of 80 % and 100 %, respectively (Morgenstern et al., 2018). Since most patients in our cohort were included before the addition of this criterion into the consensus definition, the number of PMNs/HPF was only determined in 79 fractures. Making statements based on our results should therefore be done with caution. Nonetheless, in two patients of our study group, this was the only confirmatory criteria found.

Assessing the microbiological and histopathological criteria, however, requires the collection of deep tissue cultures, which are only accessible by surgical exploration. Although a low threshold to look for confirmatory criteria is recommended when FRI is suspected, in these patients the decision to operate can solely be based on a combination of clinical, radiological and laboratory suggestive criteria (Metsemakers et al., 2018b; Govaert et al., 2020). Suggestive clinical criteria of infection (e.g., redness, swelling) were present in 123 (85 %) of the infected fractures presenting without clinical confirmatory criteria. Most of these patients presented with either one (31 %) or two (23 %) suggestive clinical criteria. This demonstrates the difficulty in diagnosing an infection in these patients and warrants a high index for suspicion when any of these criteria are present. Contrary to all other clinical suggestive criteria, pain was more prevalent in the subgroup without clinical confirmatory criteria (56 % vs. 47 %). However, pain can have multiple causes other than FRI and has shown to have a low diagnostic performance for infected fractures (Onsea et al., 2022).

Conventional radiology and CT are generally only able to detect criteria that are not specific to infection (e.g., failure of progression of bone healing), which limits their diagnostic value (Bosch et al., 2020; Onsea et al., 2022). Radiological criteria were more prevalent in the subgroup of patients presenting without clinical confirmatory criteria. A possible explanation for this is that the diagnosis of FRI is often delayed in these patients, which allows the radiological criteria to develop over a longer period of time. While MRI has potential benefits when compared to conventional radiography and CT, it was only used in eight cases of the total FRI population.

Previous research has shown a high diagnostic accuracy for nuclear imaging techniques (Bosch et al., 2020; Zhang et al., 2021). Although the WBC scan, for example, has been shown to reach a specificity of 97 % (Govaert et al., 2018), nuclear imaging is still only valid as a suggestive criterion for FRI (Zhang et al., 2021; Govaert et al., 2020). In our study, these diagnostic modalities were used in 57 FRIs, of which, 24 presented without clinical confirmatory criteria. The prevalence of any nuclear imaging criterion in this group was 71 %. ${}^{18}$ F-FDG-PET displayed criteria of infection most often. However, due to the small sample size, these results should be interpreted with caution.

Laboratory markers used for the diagnosis of FRI include CRP, WBC and ESR. The prevalence of an elevation in any of these markers was the same in all subgroups (97 %). In the subgroup of patients without clinical confirmatory criteria, an elevated CRP or WBC was present in 101 (83 %) and 48 (40 %) of the infected fractures, respectively. These results are in line with a recent systematic review, showing a sensitivity ranging from 60 %–100 % and 23 %–73 % for CRP and WBC, respectively (Van den Kieboom et al., 2018). While an elevated CRP thus has a higher sensitivity than an elevated WBC, the increase can also have other causes than infection, resulting in a lower specificity (Sigmund et al., 2020). Specifically, a persistently high CRP after the first few days postoperatively or an increase after an initial decrease should raise suspicion for FRI (Neumaier and Scherer, 2008; Metsemakers et al., 2018b)

### 4.2 Microbiological epidemiology

Antibiotics are an integral part of both the prevention and treatment of FRI (Zalavras, 2017; Depypere et al., 2020). Knowledge of microbiological epidemiology is crucial for optimizing antibiotic administration. In our study, a significant number of infections was polymicrobial (36 %). This is in line with previous studies showing a polymicrobial infection rate of approximately 30 % (Kuehl et al., 2019; Wang et al., 2021). Monomicrobial infections were mostly caused by *S. aureus* (31 %), *S. epidermidis* (9 %) and Enterobacterales (8 %) (Fig. 2.). *S. aureus* possesses multiple mechanisms to colonize bone and is widely viewed as the predominant pathogen in FRI (Moriarty et al., 2022; Masters et al., 2022). Recent research on the time-dependent microbiological epidemiology of FRI has shown an important role for Enterobacterales in acute/early infection. Delayed and chronic/late-onset infections are more often caused by less virulent organisms such as coagulase-negative Staphylococci (CNS) (Kuehl et al., 2019; Depypere et al., 2022).

### 4.3 Limitations

This study has several limitations. The lack of a control group impedes the assessment of the diagnostic accuracy of the suggestive criteria of infection. However, the study does provide an overview of the prevalence of these criteria in a large populations of FRI patients. Second, this is a retrospective study that is subject to information bias due to missing data or misclassification of data from medical files. To minimize errors in data collection, medical files were reviewed by multiple authors. Third, due to the lack of a gold standard for the diagnosis of FRI, patients were included based on the treatment they received based on recommendations of a multidisciplinary team. However, 98 % of all included patients presented with at least one confirmatory criterion. These criteria were recently proven to be pathognomonic for infection (Onsea et al., 2022). Lastly, patients were included over a relative wide period of time and in hospitals from four different countries. This is accompanied with changes in daily clinical practice and different preferences in diagnostic protocols. Consequently, some diagnostic modalities were only used in a small number of patients, which makes it hard to draw conclusion about their diagnostic value.

## 5 Conclusions

This multicenter, retrospective cohort study displays the diagnostic characteristics of 609 patients who were treated for FRI. Clinical confirmatory criteria were absent in 23 % of the FRIs. In these cases, the decision to operatively collect deep tissue cultures was based on a set of clinical, radiological and laboratory suggestive criteria. The prevalence of any criterion within these categories was 85 %, 55 % and 97 %, respectively. The combined use of these criteria should guide physicians in the management pathway of FRI. Further research is needed to provide guidelines on the decision to perform additional invasive testing when only these suggestive criteria are present.

## Data Availability

All raw data can be provided by the corresponding authors upon request.
